# Antitumor and immunomodulatory activities of total flavonoids extract from persimmon leaves in H_22_ liver tumor-bearing mice

**DOI:** 10.1038/s41598-018-28440-8

**Published:** 2018-07-12

**Authors:** Li Chen, Yundong Wei, Shimei Zhao, Mengliang Zhang, Xiaoting Yan, Xiangyun Gao, Jinxia Li, Yutong Gao, Anwen Zhang, Ying Gao

**Affiliations:** 1 Department of Pharmacology, College of Medicine, Guangxi University of Science and Technology, Liuzhou, Guangxi 545006 China; 2 Department of Chemistry, Middle Tennessee State University, Murfreesboro, TN 37132 USA; 3 School of Agriculture, International Ginseng Institute & Tennessee Center for Botanical Medicine Research, Middle Tennessee State University, Murfreesboro, TN 37132 USA

## Abstract

Persimmon (*Diospyros kaki* L.) leaves are commonly used in Asia as tea infusion and as an agent in traditional medicine. The present study aims to explore the antitumor and immunomodulatory effects of total flavonoids extract from persimmon leaves (PLF) in H_22_ liver tumor-bearing mice. We found that the PLF showed significant inhibition on the liver tumor growth in mice with a tumor inhibition rate of up to 49.35%. In contrast to the severe side effects of cyclophosphamide (CTX), the PLF exhibited anti-cachexia effect and showed no alternation in the body weight and food intake in mice. Moreover, compared with the vehicle control and CTX group, the PLF significantly enhanced the thymus and spleen indices, level of serum interleukin-18 (IL-18), monocyte/macrophage phagocytosis, level of serum hemolysin, and activity of natural killer (NK) cells. This study demonstrated that the PLF could effectively inhibit liver tumor growth *in vivo* via enhancement of the immune function in mice, and it displayed the potential to be a safe and effective anticancer agent or functional immune-enhancing agent.

## Introduction

Hepatocellular carcinoma (HCC) is one of the most common malignant tumors in humans. The mortality rate of HCC is exceeded only by that of gastric cancer and lung cancer, making it the third leading cause of cancer death worldwide^1,2^. Current chemotherapeutic agents used to treat HCC mainly focus on attacking and killing the rapidly proliferating liver cancer cells and often overlook the impact on the body’s immune response. Due to their low selectivity chemically synthesized chemotherapeutic agents cause damage to healthy organs and the immune system while killing the cancer cells^3^. Accumulating clinical and experimental studies also have shown that most cancer patients suffer from suppressed immune function coincident with the proliferation of cancer cells. These studies also demonstrate that the administration of chemotherapeutic agents aggravates the damage on the immune function resulting in serious adverse effects^4^. Therefore, there is an urgent need to develop a novel agent that can protect or enhance the body’s immune function while effectively treating HCC.

Plant-derived natural products are beneficial to ameliorate disease symptoms with less adverse effects due to their multiplex targets^5^. Previous studies have reported that a herbal prescription and plant-derived polysaccharides enhanced the body’s immune function while inhibiting tumor growth via promoting the proliferation of immune cells, accelerating the phagocytosis of macrophages, and regulating the production of cytokines^6,7^.

Persimmon (*Diospyros kaki* L.) leaves are commonly used in Asia as tea infusion and as an agent in traditional medicine. The main constituents found in persimmon leaves include flavonoids, terpenoids, naphthoquinones, coumarins, tannins, sterols, organic acids, fatty acids, and volatile oil^8^. The reported beneficial pharmacological effects of persimmon leaves include antitumor^9^, hypoglycemic^10^, antioxidant^11^, anti-inflammatory^12^, anti-bacterial^13^, antihypertensive^14^, anti-hyperlipidemia^15^, and cardiovascular protective^16^ activities. Our previous studies indicated that the ethyl acetate extract of persimmon leaves (2 g/kg) possessed antitumor activity against H_22_ hepatoma and S_180_ Sarcoma with a tumor inhibition rate of 50.5% and 38.2%, respectively^17^. Meanwhile, the thymus and spleen indices were elevated by the ethyl acetate extract of persimmon leaves. As flavonoids are the main bioactive constituents in the ethyl acetate extract of persimmon leaves, we hypothesize that the total flavonoids extract from the persimmon leaves may contribute to the antitumor and immunomodulatory activities of the ethyl acetate extract from the persimmon leaves. Recent studies reported *in vitro* proliferative and *in vivo* antitumor activities of flavonoids extracted from persimmon leaves against lung and prostate cancers^9,18^. As to our knowledge, there is no report on the cancer immunomodulation effect of total flavonoids extract of persimmon leaves (PLF) and there is no report of its efficacy against liver cancer. This study aims to investigate the *in vivo* antitumor and immunomodulatory effects of the PLF using an H_22_ tumor-bearing mice model, and therefore provide a theoretical foundation for the development of PLF as a potential immunomodulatory agent that can help to reverse the immune deficiency on cancer patients.

## Results

### Rutin, quercetin, myricitrin, kaempferol, and myricetin represented the major flavonoids in the PLF

**Figure 1:**
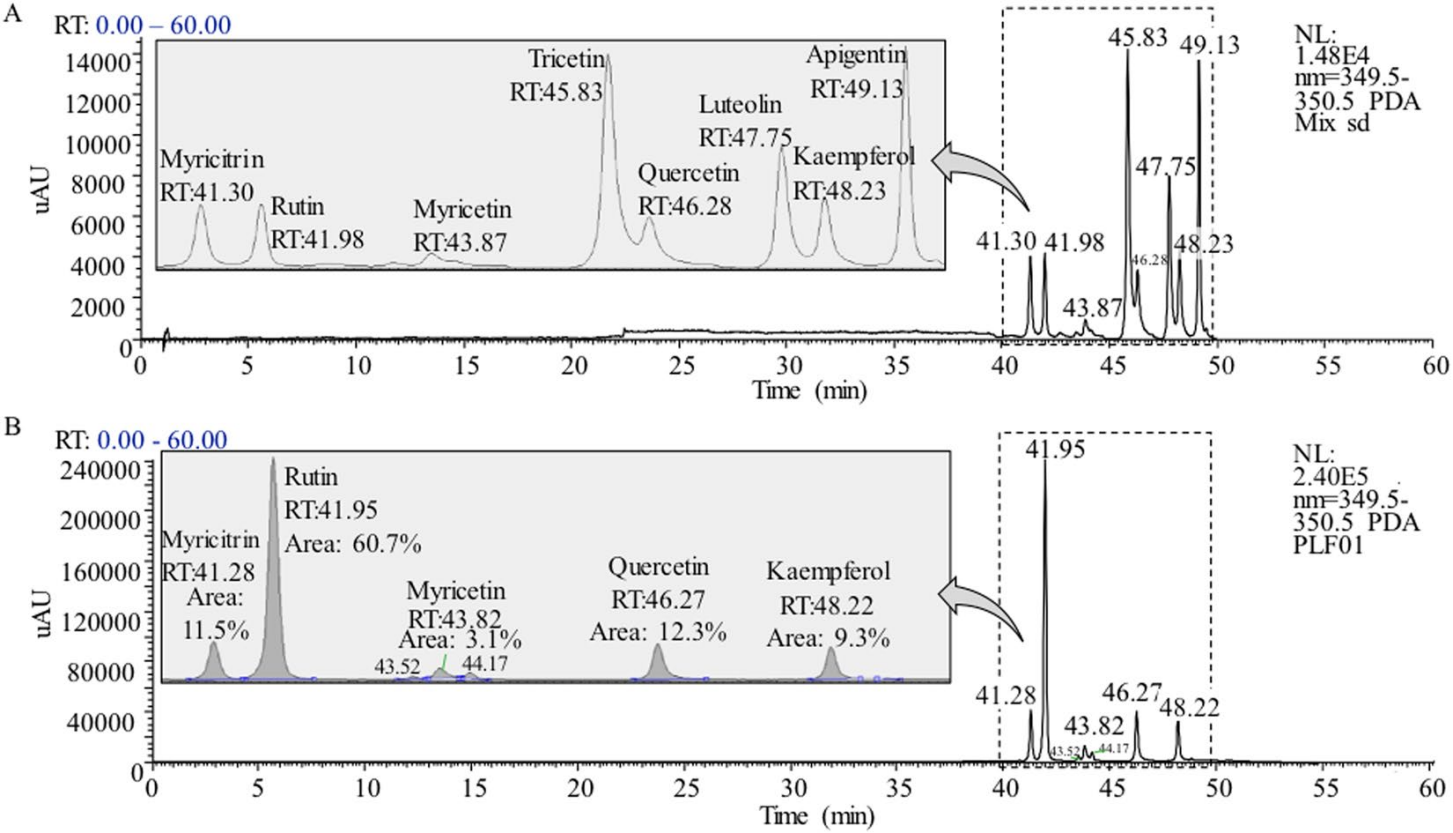
UHPLC-UV-Vis chromatograms at 350 nm for (**A**) eight flavone and flavonol glycosides reference standards and (**B**) the PLF.

Persimmon leaves are known to contain high levels of flavone and flavonol glycosides, which have characteristic UV-Vis absorbance at around 354 nm^19^; therefore 354 nm was used as the main acquisition wavelength in the UHPLC-PDA analysis. Eight flavone and flavonol glycoside reference standards and the PLF extract was analyzed by using UHPLC-PDA-ESI/MS^n^ method, and their representative UV-Vis chromatograms at 354 nm are shown in Fig. 1. In PLF sample, eight peaks were observed and five of them were identified as myricitrin, rutin, myricetin, quercetin, and kaempferol by comparing retention times, UV-Vis spectra, and MS^n^ spectra with reference standards (Fig. 1B). Lin *et al*. previously reported the molar relative response factors (MRRF) of most common flavone and flavonol glycosides to rutin at 354 nm, and concluded that MRRF of 1.0 at UV 354 nm could be used to determine the quantities of flavone and flavonol glycosides, with the exception of hinokiflavone (MRRF = 1.94) and cupressuflavone (MRRF = 1.94)^20^. In this study, an assumed MRRF of 1.0 at UV 354 nm was used to quantitate the flavone and flavonol glycosides. The molar percentage of individual flavone and flavonol glycoside in the total flavone and flavonol glycosides was calculated by comparing its corresponding peak area to the total peak areas at 354 nm. The five identified flavone and flavonol glycosides represented 96.9% of total flavone and flavonol glycosides in the PLF, with 60.7% of rutin, 12.3% of quercetin, 11.5% of myricitrin, 9.3% of kaempferol, and 3.1% of myricetin, respectively.

### PLF suppressed the H_22_ solid liver tumors in mice

**Figure 2:**
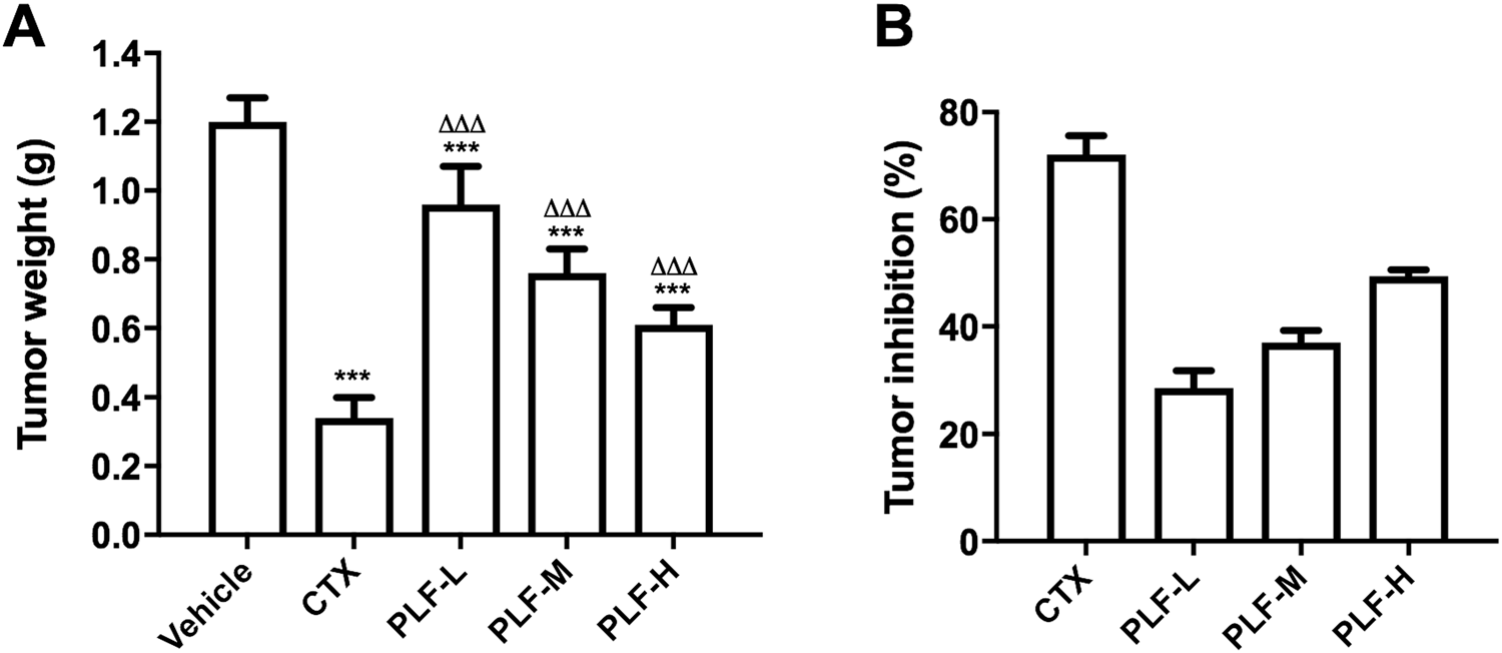
Tumor inhibitory effects of the PLF in mice bearing H_22_ solid tumors. After i.p. CTX or PLF for ten days, the tumors were excised and weighed, and tumor inhibition rates were calculated. (**A**) Effect of the PLF on tumor weight, (**B**) Effect of the PLF on tumor inhibition rate. Data were presented as means ± standard deviation (n = 10). ***p < 0.001, compared with the vehicle group; ^∆∆∆^*p* < 0.001, compared with the CTX group.

We assessed the *in vivo* antitumor effect of the PLF using H_22_ tumor-bearing mice as an animal model. The tumor weights and tumor inhibition rates were shown in Fig. 2. The vehicle control showed an average tumor weight of 1.20 ± 0.07 g when the tumors were excised and weighed at the 11^th^ day. In contrast, the treatment of CTX and PLF effectively suppressed the tumor growth (***p < 0.001). The average tumor weight of CTX treatment group was 0.34 ± 0.06 g, resulting in a tumor inhibition rate of 72.1% compared with the vehicle control. The average tumor weights of the PLF-L, -M, and -H treatment groups were 0.96 ± 0.11 g, 0.76 ± 0.07 g, and 0.61 ± 0.05 g, respectively, resulting in tumor inhibition rate of 28.59%, 37.09%, and 49.35%. These results also suggest that the PLF displayed a dose-dependent efficacy.

### Effect of the PLF on the histological features of H_22_ solid liver tumors

**Figure 3:**
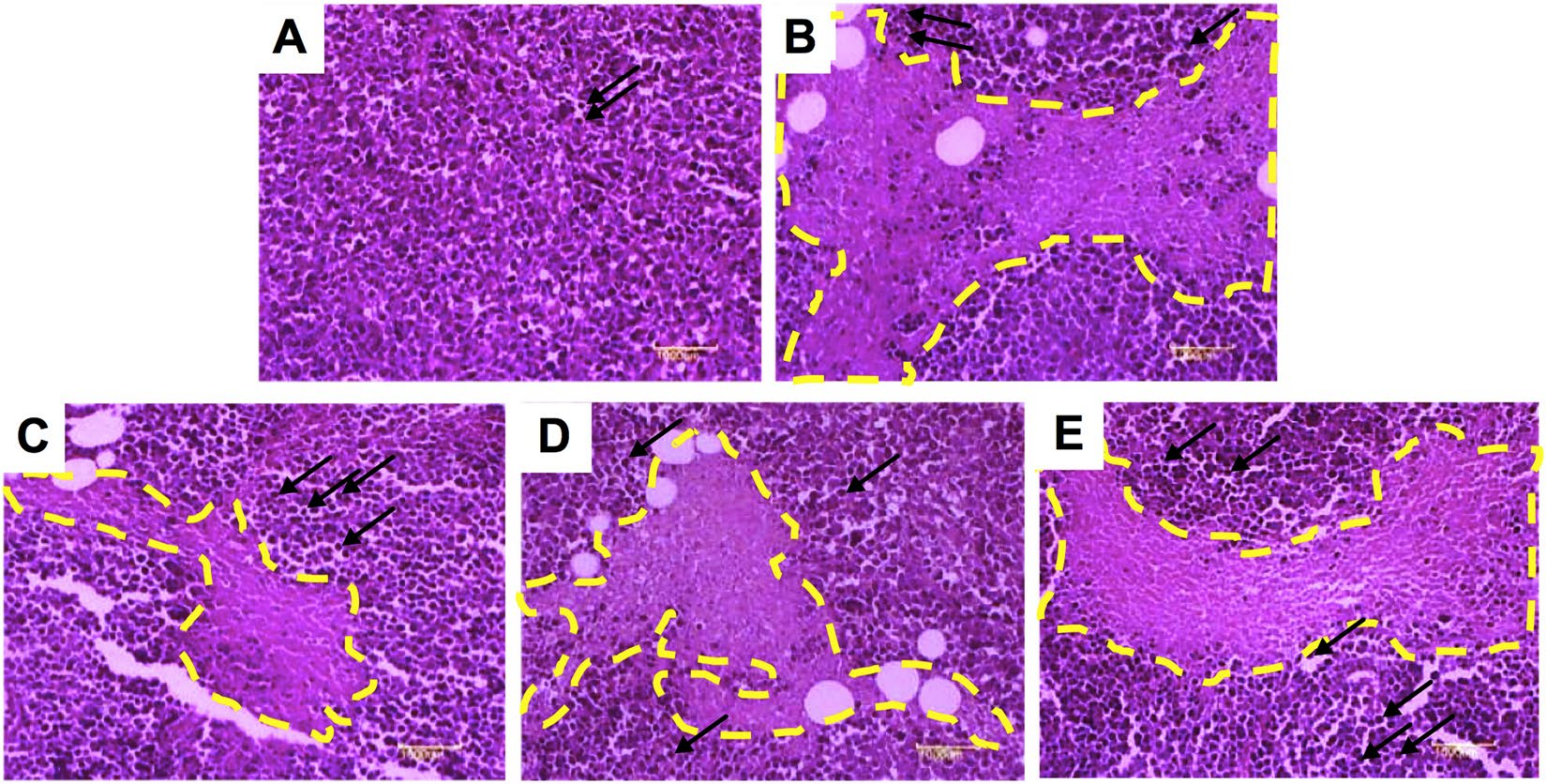
Histological examination of tumor tissues from H_22_ tumor-bearing mice. The tumors were sectioned and stained with hematoxylin–eosin (H&E). The nuclei were indicated in dark purple, the necrotic tumor foci were marked with yellow dotted lines, and the lymphocyte infiltrations were indicated by the black arrows.(**A**) The vehicle group, (**B**) The CTX group, (**C**) The PLF-L group, (**D**) The PLF-M group, (**E**) The PLF-H group. Magnification: 100x.

To further investigate the antitumor efficacy of the PLF, we performed histological analysis of the excised tumors from the mice at the 11^th^ day. As shown in Fig. 3A, the tumor cells from the mice in the vehicle group were tightly arranged and distributed diffusely. The tumor cells showed large nucleus or double nuclei (indicated by dark purp spots), and clearly apparent nucleolus. However, in CTX (Fig. 3B) and PLF (Fig. 3C,D,E) treatment groups, the tumor sections exhibited notably different histological features. Large areas of necrotic tissues, nuclear shrinkage, and decreased percentage of nuclei in tumor cells were observed in the CTX and PLF groups. A high percentage of the necrosis area and a small percentage of remaining tumor cells were observed in the CTX group. Moreover, the percentage of necrosis area in PLF-L, -M, and -H treatment groups suggest a dose-dependent antitumor efficacy by the PLF. Visible scattered lymphocyte infiltrations were observed in the CTX group (indicated by the black arrows). Compared with the vehicle and CTX groups, more scattered or aggregated lymphocyte infiltrations were observed in the PLF groups, suggesting that the PLF enhanced the response of the body’s immune system to tumor tissue in mice. These results further confirmed the antitumor and immune-enhancing efficacy of the PLF.

### Effect of the PLF on food-intake and the body weight of H_22_ tumor-bearing mice

The body weight and food intake of mice were measured to evaluate the side effects of the treatments (Tables 1 and 2). After inoculation of the ascitic tumor cells, the mice displayed less food-intake, loss of body weight, hair loss, and cachexia, which indicated the consequences of the H_22_ liver tumor growth. Average body weights of all groups showed no significant difference at day 1 (*p* > 0.05). At days 4–11, the average body weights of the vehicle group were significantly lower than that of the naïve group (^##^*p* < 0.01 or ^###^*p* < 0.001), despite the increases of the tumor weights. During CTX administration, the body weights of mice continuously decreased, and were significantly lower compared with the naïve group (^##^*p* < 0.01 or ^###^*p* < 0.001) as well as the vehicle control group (^***^p < 0.001) at days 4–11. At day 11, the average body weight of CTX treated mice was even lower than day 1. CTX treated mice also displayed server cachexia and hair loss. Notably, at day 9 two mice in the CTX treatment group died. Prior to their death, similar symptoms as late stage liver cancer patients were observed, including asthma, difficulty in breathing, dramatic weight loss, and loss of appetite. However, in contrast to the CTX group, the body weights of mice in all PLF treatment groups (PLF-L, -M, and -H) steadily increased (^∆^*p* < 0.05 or ^∆∆∆^*p* < 0.001) and no significant difference was observed in all PLF treatments groups (PLF-L, -M, and -H) compared with the naïve groups (*p* > 0.05) except PLF-M at day 7.

**Table 1:** Effect of the PLF on the body weight in H_22_ tumor-bearing mice.

Group	Death during treatment	The body weight (g)			
		Day 1	Day 4	Day 7	Day 11
Naïve	0	21.27 ± 1.65	26.39 ± 1.75	30.33 ± 1.65	32.79 ± 1.50
Vehicle	0	21.08 ± 0.83	22.32 ± 2.15^##^	26.54 ± 1.85^###^	29.27 ± 2.30^###^
CTX	2	20.82 ± 1.43	23.55 ± 1.54^##^	20.57 ± 1.74^###^***	18.65 ± 1.84^###^***
PLF-L	0	21.73 ± 1.09	24.83 ± 1.56*	28.31 ± 1.96^∆∆∆^	31.67 ± 1.22^∆∆∆^
PLF-M	0	21.63 ± 1.29	25.82 ± 2.62*^∆^	27.83 ± 2.61^#∆∆∆^	31.35 ± 2.87^∆∆∆^
PLF-H	0	21.82 ± 1.07	23.94 ± 4.12	29.43 ± 1.30***^∆∆∆^	32.34 ± 1.97**^∆∆∆^

Data were presented as means ± standard deviation (n = 10). ^#^*p* < 0.05, ^##^*p* < 0.01, ^###^*p* < 0.001, compared with the naïve group; *p < 0.05, **p < 0.01, ***p < 0.001, compared with the vehicle group; ^∆^*p* < 0.05, ^∆∆^*p* < 0.01, ^∆∆∆^*p* < 0.001, compared with the CTX group.

**Table 2:** Effect of the PLF on food intake in H_22_ tumor-bearing mice.

Group	Food intake (g)			
	Day 1	Day 4	Day 7	Day 11
Naïve	34.68	36.62	41.77	37.22
Vehicle	32.29	28.61	26.42	20.84
CTX	30.22	18.61	14.03	7.37
PLF-L	30.63	30.6	34.24	33.31
PLF-M	29.79	31.08	33.46	32.87
PLF-H	29.03	32.44	38.59	35.3

Similarly, decreases in food intake were observed in the vehicle and CTX groups at days 4–11 (Table 2). Notably, the average food intake of mice in the CTX group was dramatically reduced from 30.22 g to 7.37 g. However, food intake in all PLF treatment groups was greater compared with vehicle control and the CTX group, and is similar to the naïve group.

Additionally, PLF treatments also remarkably improved cachexia of tumor-bearing mice. Notably, mice displayed normal activity in the PLF-H group. In all PLF treatment groups, there was no observable hair loss in mice. These data clearly suggest that the PLF does not cause side effects and has an anti-cachexia effect in mice.

### Effect of the PLF on the immune organs of H_22_ tumor-bearing mice

**Figure 4:**
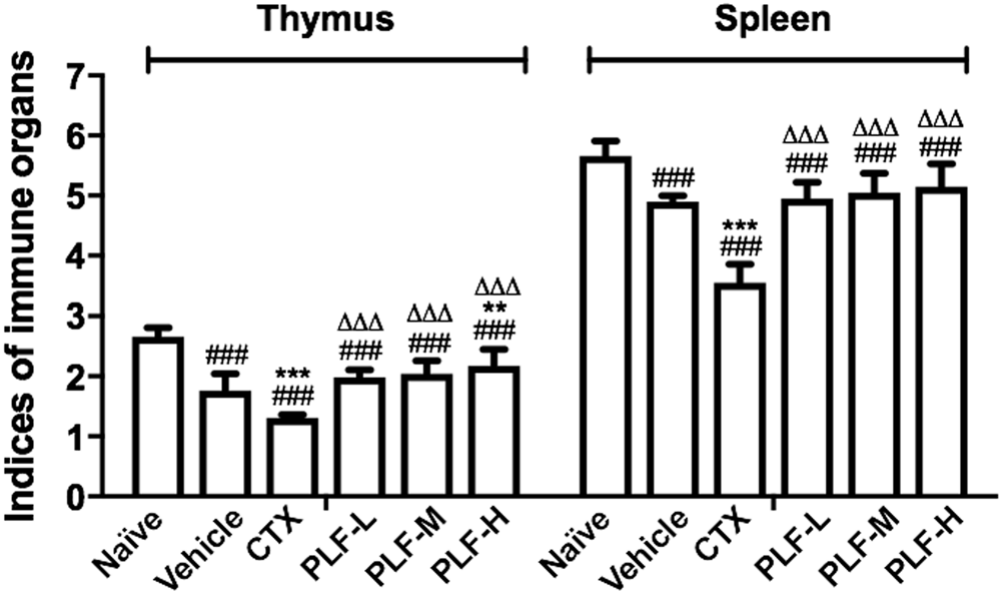
Effect of the PLF on thymus and spleen indices in H_22_ tumor-bearing mice. Spleen or thymus indices were measured in the ratio of the spleen or thymus weight (mg) to the body weight (g) of each mouse. Data were presented as means ± standard deviation (n = 10). ^###^*p* < 0.001, compared with the naïve group; **p < 0.01, ***p < 0.001, compared with the vehicle group; ^∆∆∆^*p* < 0.001, compared with the CTX group.

We examined the effects of the PLF on the immune organs of H_22_ tumor-bearing mice including spleen and thymus. As shown in Fig. 4, the thymus and spleen indices in the vehicle control are significantly lower compared with the naïve group (^###^*p* < 0.001). After CTX administration, both the spleen and thymus indices were significantly lower compared with the vehicle control (^***^p < 0.001) and the naïve group (^###^*p* < 0.001), suggesting that the immune function of the mice was remarkably suppressed in the H_22_ tumor-bearing mice and further suppressed by CTX. However, after administration with PLF-L, -M, or -H, the spleen indices were increased by 12.5%, 15.9%, and 23.3%, respectively, compared with that of the vehicle control (^**^p < 0.01). Additionally, PLF administration also slightly increased the thymus indices in mice compared with the vehicle group, although no significant difference was found (*p* > 0.05). The results suggest that PLF stimulated thymus and spleen development in a dose-dependent manner. Moreover, compared with the CTX, both thymus and spleen indices were significantly increased by the PLF at all three dosages (^∆∆∆^*p* < 0.001).

### Effect of the PLF on the serum level of IL-18 in H_22_ tumor-bearing mice

**Figure 5:**
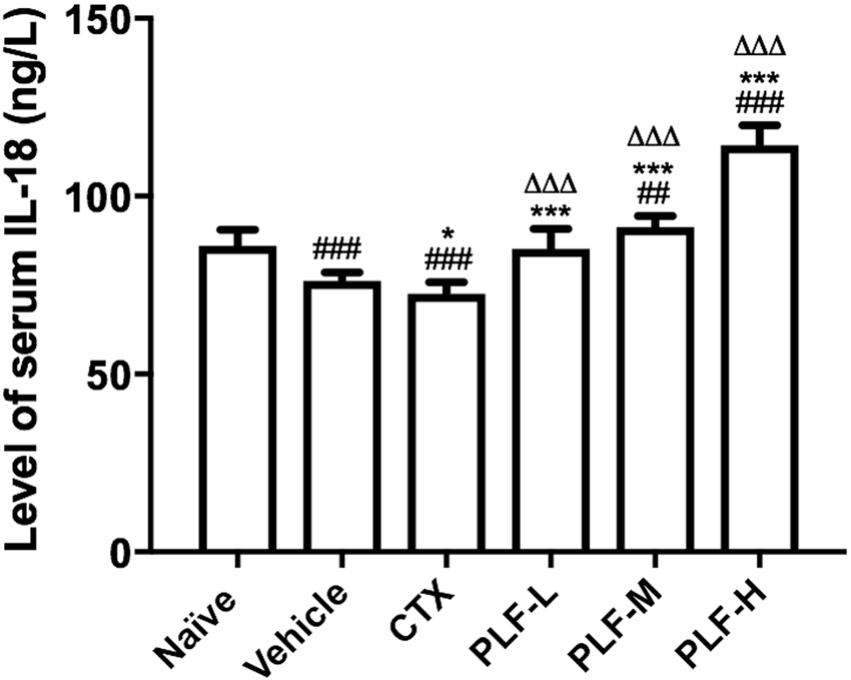
Effect of the PLF on the level of serum IL-18 in H_22_ tumor-bearing mice. The level of IL-18 in mice serum was quantified using a Mouse IL-18 ELISA kit. Data were presented as means ± standard deviation (n = 10). ^###^*p* < 0.001, compared with the naïve group; *p < 0.05, ***p < 0.001, compared with the vehicle group; ^∆∆∆^*p* < 0.01, compared with the CTX group.

We investigated the effect of the PLF on the immunity-related cytokine IL-18. As shown in Fig. 5, the secretion of IL-18 in the vehicle group and the CTX group decreased significantly compared with the naïve group (^###^*p* < 0.001). Moreover, the level of IL-18 in the CTX group further decreased compared with the vehicle group (*p < 0.05). However, the production of IL-18 in all PLF groups was significantly upregulated compared with that of the vehicle group (***p < 0.001). The PLF-L, -M, and –H elevated the level of IL-18 by 11.7%, 19.6%, and 50.0%, respectively. The PLF-L restored the serum IL-18 to the normal level, and more strikingly, the PLF-M and -H significantly upregulated the serum IL-18 compared with that of the naïve control (^##^*p* < 0.01 or ^###^*p* < 0.001).

### Effect of the PLF on the phagocytosis of monocyte/macrophage in H_22_ tumor-bearing mice

**Figure 6:**
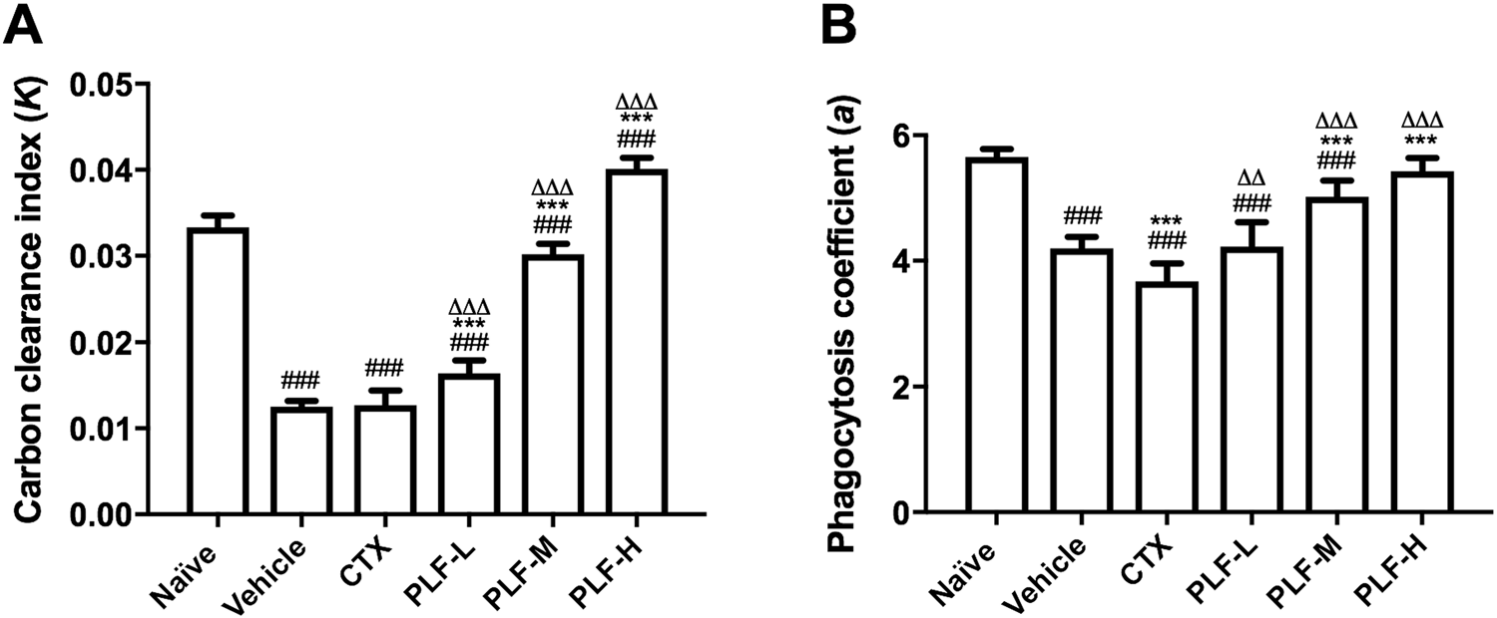
Effect of the PLF on the phagocytosis of monocyte/macrophage in H_22_ tumor-bearing mice. After i.p. CTX or PLF for ten days, India ink was injected into the mouse, and blood was collected and measured for optical density to calculate the carbon clearance index. The liver and spleen were weighed to calculate the phagocytosis coefficient. (**A**) Effect of the PLF on the carbon clearance index (*K*). (**B**) Effect of the PLF on the phagocytosis coefficient (*a*). Data were presented as means ± standard deviation (n = 10). ^###^ *p* < 0.001, compared with the naïve group; ***p < 0.001, compared with the vehicle group; ^∆∆^*p* < 0.01, ^∆∆∆^*p* < 0.001, compared with the CTX group.

To evaluate the monocyte/macrophage phagocytic function, we conducted a carbon clearance test. The elimination rate of particulate carbon that was intravenously injected into the mouse reflects the monocyte/macrophage phagocytosis^21^. As shown in Fig. 6A, the carbon clearance index (*K*) in the vehicle control group and the CTX treatment group were significantly lower than that in the naïve control group (^###^*p* < 0.001). In comparison with the vehicle group, PLF treatment at all dosages remarkably improved the carbon clearance index in mice (***p < 0.001). Moreover, the PLF-H even displayed a higher carbon clearance index than the naïve control (^###^*p* < 0.001).

Similarly, the phagocytosis coefficient (*a*) in the vehicle control group and the CTX treatment group were significantly lower than that in the naïve control group (^###^*p* < 0.001) (Fig. 6B). Although the phagocytosis coefficients in the PLF-M and -H groups were still lower than that of the naïve control group, they were significantly enhanced compared with the vehicle control (***p < 0.001). PLF-L group exhibited slightly higher phagocytosis coefficients than the vehicle control but no statistical significance was observed. Moreover, compared with the CTX treatment group, all of the PLF-treated groups exhibited significantly higher carbon clearance index and phagocytosis coefficient (^∆∆^*p* < 0.01 or ^∆∆∆^*p* < 0.001). These results suggest that the decline in the monocyte/macrophage phagocytic function in the H_22_ tumor-bearing mice were reversed by the PLF administration.

### Effect of the PLF on the production of serum hemolysin in H_22_ tumor-bearing mice

**Figure 7:**
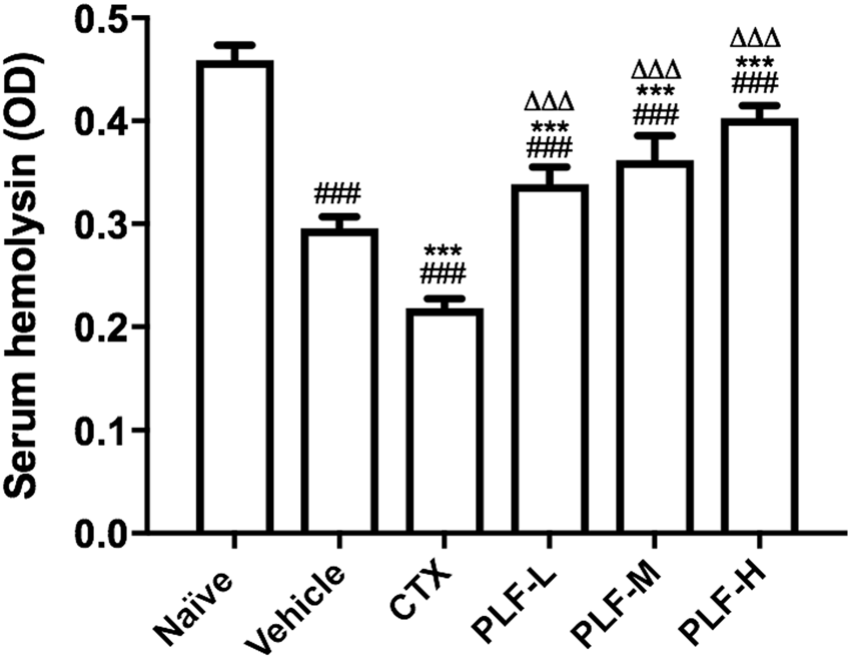
Effect of the PLF on the production of serum hemolysin in H_22_ tumor-bearing mice. On the fifth day of drug administration, chicken red blood cells were injected into each mouse for five continuous days. Blood was collected and serum hemolysin was measured. Data were presented as means ± standard deviation (n = 10). ^###^*p* < 0.001, compared with the naïve group; ***p < 0.001, compared with the vehicle group; ^∆∆∆^*p* < 0.001, compared with the CTX group.

To investigate the effect of the PLF on the immunosuppressed complement system, we measured the level of serum hemolysin produced in response to the cellular antigen. As shown in Fig. 7, the production of serum hemolysin was suppressed in the vehicle control group compared with the naïve control group (^###^*p* < 0.001). Moreover, the serum hemolysin was further decreased after the CTX administration in comparison with the vehicle control (***p < 0.001). In contrast, in all of the PLF-treated groups, the hemolysin levels were significantly higher than that of the vehicle control (***p < 0.01), although they were still lower than that of the naïve control. Moreover, compared with the CTX group, all of the PLF-treated groups exhibited a significantly elevated level of serum hemolysin (^∆∆∆^*p* < 0.001).

### Effect of the PLF on the NK cell cytotoxicity in H_22_ tumor-bearing mice

**Figure 8:**
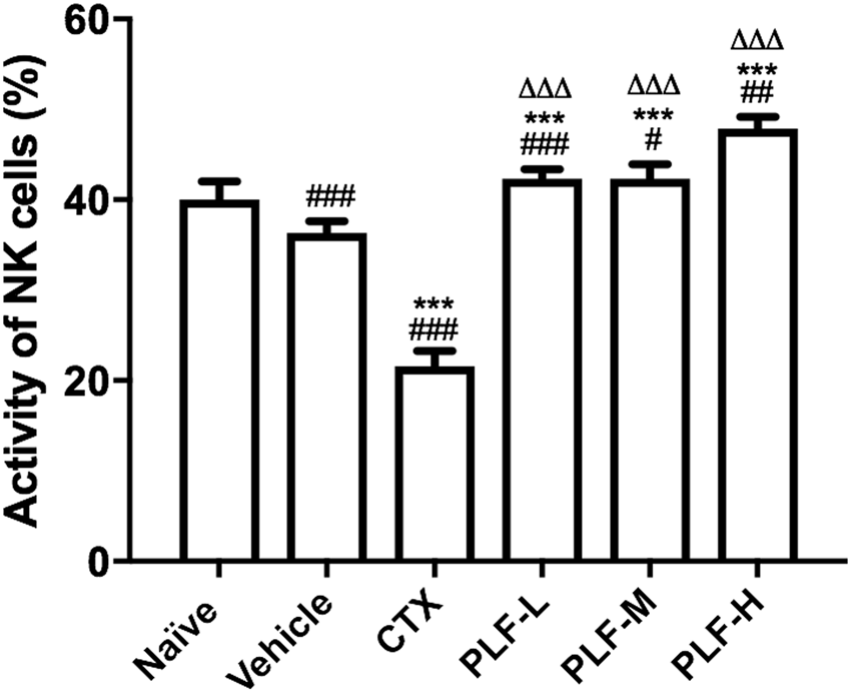
Effect of the PLF on the NK cell cytotoxicity in H_22_ tumor-bearing mice. The cytotoxic activity of the NK cells in spleen lymphocytes against YAC-1 cells was measured using a Lactate Dehydrogenase (LDH) Cytotoxicity Detection Kit. Data were presented as means ± standard deviation (n = 10). ^###^*p* < 0.001, compared with the naïve group; ***p < 0.001, compared with the vehicle group; ^∆∆∆^*p* < 0.001, compared with the CTX group.

We assessed the cytotoxic activity of NK cells in spleen lymphocytes against YAC-1 mouse lymphoma cell line, an NK-sensitive tumor cell line. The cytotoxicity was measured based on the amount of LDH that was correlated with the number of damaged YAC-1 cells. As shown in Fig. 8, the cytotoxic activity of NK cells in the vehicle control group were suppressed compared with the naïve group (^###^*p* < 0.001), and the CTX administration further suppressed the NK cell cytotoxicity compared with the vehicle control (***p < 0.001). In contrast, the PLF administration at all dosages significantly enhanced the NK cell cytotoxicity compared with the vehicle control (***p < 0.001), and restored it to the normal level or even enhanced it compared with the naïve control (^#^*p* < 0.05, ^##^*p* < 0.01, or ^###^*p* < 0.001). In comparison with the CTX treatment, the PLF treatments also exhibited significantly enhanced NK cell cytotoxicity (^∆∆∆^*p* < 0.001).

## Discussion

In this study, we identified five major flavone and flavonol glycosides in the PLF, namely rutin, quercetin, myricitrin, kaempferol, and myricetin. Our finding is consistent with the constituents in the extract of persimmon leaves reported in the previous reports^8^. The five compounds have been reported to possess antitumor efficacy against a variety of cancer cell lines. Rutin is known to have inhibitory activity against leukemia, colorectal cancer, neuroblastoma, and melanotic melanoma cell lines^22^. Quercetin was shown to effectively inhibit prostate cancer both *in vitro* and *in vivo*^23^. Myricitrin and myricetin have been found to show *in vitro* inhibitory effect against human prostate cancer cell line^24^. Kaempferol was reported to inhibit breast cancer, ovarian cancer, bone cancer, leukemia cells by modulating apoptosis, angiogenesis, inflammation, and metastasis^25^. Some researchers considered that isolation of a single active compound from a plant-based food might compromise its therapeutic efficacy due to the importance of synergistic interaction among compounds^26^. Several studies highlighted the synergistic antitumor effects among the flavonoid compounds of persimmon leaves extracts. Ding *et al*.^18^ reported that the total flavonoid compounds from persimmon leaves showed a higher anti-proliferative and apoptosis induction efficacy in PC-3 prostate cancer cells compared with Quercetin and Rutin alone^18^. Another study demonstrated that the total flavonoid compounds isolated from persimmon leaves showed superior antioxidant activity than Rutin alone^11^. These studies implied that the better effectiveness of the total flavonoid extract might be consequent to the combination of the multiple flavonoids in the extract. In our study, the antitumor and immune-enhancing efficacy of total flavonoids extract of persimmon leaves is possibly benefited from the combination of the flavonoids.

Cyclophosphamide (CTX) is used as a broad-spectrum anticancer drug as well as a cytotoxic immunosuppressive drug. It is currently one of the most widely prescribed immunosuppressive drugs and is also frequently used to establish immunosuppressed animal models^27^. CTX is known to suppress the immune system via myelosuppression and toxicity towards lymphocytes^28^. In this study, CTX was selected as a positive control. We found that CTX (0.12 g/kg) remarkably inhibited the growth of liver tumors in mice with an inhibition rate of 72.10% and induced major necrosis in the tumor tissues, confirming that CTX is an effective antitumor agent against H_22_ liver tumors. However, at the same time CTX also induced significant immunosuppression and severe side effects. Compared with the naïve group and the vehicle group, CTX significantly decreased the thymus and spleen indices, serum hemolysin, NK cell cytotoxicity and serum IL-18 in H_22_ tumor-bearing mice, suggesting that CTX suppressed the overall immune function of the mice. During the CTX treatment, the food intake of mice remarkably decreased, resulting in serious loss of body weight compared with the naïve and vehicle controls. Several visual symptoms including unusual weakness, severe hair loss, lethargy, as well as a 20% death rate were observed during CTX treatment. These observations are consistent with the previously reported adverse effects of CTX^29^. In contrast, PLF administration in all three doses did not alter food intake or body weight of the mice and they displayed normal activity with no observable hair loss. PLF treatments also remarkably alleviated cachexia of tumor-bearing mice and restored their normal activity. These results demonstrate that PLF did not cause adverse effects and had an anti-cachexia effect in mice.

Thymus and spleen are key organs of the immune system. The thymus is the major site of T cell differentiation, development, and maturation and is closely related to humoral immunity. The spleen plays an essential role in the synthesis of immune cells and monocytes. Therefore, thymus and spleen indices reflect the status of the body’s immune function. In this study PLF at all dosages significantly elevated the thymus and spleen indices compared with the vehicle control and CTX, suggesting the protective effect of the PLF on the immune organs in mice.

Natural killer (NK) cells are lymphocytes that are one of the main effectors of immunity. NK cells exert tumor immune surveillance function via direct cytotoxic activity against cancer cells and/or secretion of cytokines and chemokines to eliminate the cancer cells in the body^30^. Recent studies have demonstrated that the reduced number and activity of NK cells in cancer patients is a key factor in tumor immune escape and is closely related to the occurrence and development of cancer^31^. The cytotoxic activity of NK cells is regulated by cytokines, such as IL-18, a multifunctional cytokine that is produced by macrophages. IL-18 induced the secretion of IFN-γ, IL-2, GM-CSF, TNF-α by the NK cells, and these secreted cytokines exert antitumor effects by directly killing tumor cells or regulating the immune function^32,33^. We found that PLF at all three dosages promoted the level of serum IL-18 and NK cell cytotoxicity, indicating that PLF induces IL-18 production, possibly by stimulation of macrophages, thereby upregulating the NK cell-mediated antitumor immune response.

Phagocytes are important immune cells whose immune function is to ingest pathogens and abnormal cells. Phagocytes are considered to be the first line of defense of the body’s immune response^34^. Monocytes/macrophages are the most important type of phagocytes and one of the crucial constituents of nonspecific immunity. Previous studies have demonstrated that decline in the phagocytic activity of monocyte/macrophage leads to the occurrence of tumors while the enhancement of monocyte/macrophage phagocytosis suppresses the growth and development of tumors^35^. In this study, we demonstrated that PLF at all three dosages significantly improved the carbon clearance index (K) and PLF at medium and high dosages significantly improved the phagocytosis coefficient (*a*). These results indicate that PLF promotes the phagocytic activity of monocyte/macrophage.

Chicken red blood cells (CRBC) are a strong antigen to mice and immunization with CRBC results in the production of a specific antibody in serum, hemolysin. The release of hemolysin to the peripheral blood leads to the hemolysis response against CRBC^36^. The level of serum hemolysin is a major indicator for nonspecific immune function, which reflects the proliferation and differentiation of B cells and their ability to secrete hemolysin after binding to the complement. PLF at all three dosages significantly enhanced the level of serum hemolysin, implying that PLF stimulated antibody-secreting B cells promoting the nonspecific immune function in tumor-bearing mice.

## Conclusion

In this study, we investigated the chemical profile of the PLF and its antitumor and immunomodulatory effects in H_22_ hepatoma mice. Besides the inhibition of tumor growth and induction of tumor tissue necrosis, we also observed the promotion of immune organ indices, monocyte/macrophage phagocytosis, splenic NK cell cytotoxicity, levels of IL-18 and hemolysin. The results demonstrate that the PLF may exert its antitumor activity via enhancement of the immune function of H_22_ tumor-bearing mice. This study suggests the potential application of the natural-occurring PLF as a safe and effective immunomodulatory agent that may reverse immune suppression in cancer.

## Methods

### Chemicals and reagents

Cyclophosphamide was purchased from Baxter Oncology GmbH (Batch No. 5J078A, Halle, Germany). Guinea pig serum was purchased from Beijing Bersee Bio Co., Ltd. Mouse IL-18 ELISA kit was purchased from R&D Systems (Minneapolis, USA). RPMI 1640 medium and fetal bovine serum (FBS) were purchased from Thermo Fisher Scientific (Waltham, MA, USA). Lactate Dehydrogenase (LDH) Cytotoxicity Detection Kit was purchased from TaKaRa (Otsu, Japan). LC/MS grade formic acid and methanol were purchased from Fisher Scientific (Fair Lawn, NJ). HPLC grade water was prepared from distilled water using a Milli-Q system (Millipore Laboratory, Bedford, MA). Rutin, quercetin, tricetin, myricitrin, kaempferol, myricetin, apigenin, and luteolin reference standards were obtained from Sigma-Aldrich (St Louis, MO).

### Total flavonoid extraction from the persimmon leaves

Persimmon (*Diospyros kaki* L.) leaves were purchased from Yaoyuan Trading Inc. (Anguo City, Hebei Province, China) (Batch No. 161011). The extraction method was described by Chen *et al*. in a previous study^37^. In brief, the persimmon leaves (20 g) were powdered and passed through 40–80 mesh sieves. The powder was soaked in petroleum ether (1:15, w/v) for 12 h and extracted twice in a Soxhlet extractor. Subsequently, the petroleum ether was evaporated and the solid was mixed with 70% ethanol (1:10, w/v). The solution was sonicated at 200 kHz at 30 °C for 30 min, and extracted three times in a Soxhlet extractor. The extract solutions were combined and concentrated to yield dried crude extract. The crude extract was adjusted to pH 10 using 20% NaOH and centrifuged at 3000 rpm for 15 min. The supernatant was adjusted to pH 6 using 15% HCl and centrifuged at 3000 rpm for 15 min. Subsequently the solution was extracted with ethyl acetate and concentrated to yield dried total flavonoids extract.

### Determination of chemical profile

An UHPLC-PDA-ESI/MS^n^ system (Thermo Scientific, San Jose, CA) was used to determine the flavone and flavonol glycosides of the PLF extract. The separation was carried out on a Thermo Scientific Hypersil Gold HPLC Column (100 × 2.1 mm i.d., 1.9 µm, Hypersil Gold PFP) with an UltraLine UHPLC In-Line Filter (RESTEK, Bellefonte, PA) at a flow rate of 0.3 mL/min. The mobile phase consisted of a combination of A (0.1% formic acid in water, v/v) and B (methanol). The linear gradient was from 5 to 20% B (v/v) at 30 min, to 60% B at 45 min, and to 100% B at 50 min and held at 100% B to 55 min. The PDA was set at 225, 350, and 275 nm to record the peaks, and UV-vis spectra were recorded from 200 to 600 nm. The volume of sample injection was 10 μL.

The mass spectrometer was operated both in positive and negative ionization modes, and the conditions were set as follows: sheath gas at 35 (arbitrary units), auxiliary and sweep gas at 15 (arbitrary units), spray voltage at 4.5 kV, capillary temperature at 500 °C, capillary voltage at 10 V, and tube lens at 100 V. The mass range was from *m/z* 150 to 1000. The most intense ion was selected for the data-dependent scan to offer their MS2 product ions with normalization collision energy at 35%.

### Cell culture

Mouse hepatoma 22 ascitic tumor (H_22_) was obtained from Guangxi Institute of Traditional Chinese Medicine and was maintained in the ascitic form by sequential passages in Kunming mice by weekly intraperitoneally (i.p.) transplantations of 1 × 10^7^ H_22_ tumor cells in 0.2 mL solution. Mouse T lymphoma cell line YAC-1 was provided by the School of Pharmacy at Guangxi Medical University. YAC-1 cells were cultured in RPMI 1640 medium supplemented with 10% FBS and 100 units/mL penicillin/streptomycin under a humidified atmosphere containing 5% CO_2_ at 37 °C.

### Animal models and experimental design

All animal handling procedures in this study were conducted in accordance with the guidelines for the use and care of laboratory animals and were approved by the Experimental Animals Committee at Guangxi University of Science and Technology. Six-week-old Kunming mice (half male and half female, SPF) weighing 18–22 g, were purchased from Human SLAC Laboratory Animal Co., Ltd. (Certificate No. SCXK 2011–0003, Changsha, China).

A total of 240 mice were used in this study for four sets of experiments: 1) The first experiment was performed to evaluate the tumor inhibition rate, level of serum IL-18, thymus index, and spleen index, see **2.5–2.7**; 2) The second experiment was performed to assess the phagocytic function of monocyte/macrophage, see **2.8**; 3) The third experiment was performed to detect the production of hemolysin antibody in the serum, see **2.9**; 4) The fourth experiment was performed to assess the activity of NK cells, see **2.10**. In each experiment, 60 mice were randomly divided into the following six groups (n = 10): naïve group, vehicle group (ddH_2_O, 20 mL/kg), cyclophosphamide (CTX) group (0.12 g/kg), low-dose PLF group (PLF-L, 0.5 g/kg), medium-dose PLF group (PLF-M, 0.25 g/kg), and high-dose PLF group (PLF-H, 1.0 g/kg).

The solid tumor model was established using the method described by Ou *et al*. in a previous study^38^. Briefly, after H_22_ cells were passaged in the abdomen cavity for 7–9 days, the mouse was sacrificed and ascites were collected under sterile conditions. The ascites were diluted with sterile saline to 1 × 10^6^ cells/mL and then inoculated via subcutaneous injection at the front right armpit of mice (0.2 mL each) except the naïve group. After 24 h inoculation, the mice were treated with one of the following agents by intragastric administration daily for ten continuous days: vehicle, CTX, PLF-L, PLF-M or PLF-H.

### *In vivo* antitumor efficacy

The food intake of each mouse was recorded daily during drug administration. On days 1, 4, 7, and 11, the body weights of mice were measured. On day 11, the mice were anesthetized and killed by cervical dislocation. The eyeball blood, thymus, and spleen of each mouse were collected. The subcutaneous tissue in the front right armpit was cut open and the tumor was carefully excised and weighed. The tumor tissue was fixed in 10% Formalin for 24 h and cut into 1 mm3 slices. After staining by hematoxylin & eosin (H&E), the histological changes of tumor tissue were observed under an Olympus BX-53 microscope (Tokyo, Japan). The tumor inhibition rate was calculated based on equation (1).

1 $$ \% \,{\rm{of}}\,{\rm{tumor}}\,{\rm{inhibition}}=\frac{{\rm{tumor}}\,{\rm{weight}}\,{\rm{in}}\,{\rm{model}}\,{\rm{group}}\,({\rm{g}})-\,{\rm{tumor}}\,{\rm{weight}}\,{\rm{in}}\,{\rm{experimental}}\,{\rm{group}}\,({\rm{g}})}{{\rm{tumor}}\,{\rm{weight}}\,{\rm{in}}\,{\rm{model}}\,{\rm{group}}\,({\rm{g}})}\times 100$$

### Serum level of IL-18

The eyeball blood samples were centrifuged at 3,000 r/min at 4 °C for 15 min. The serum was collected for the detection of IL-18 level using a commercial ELISA kit according to the kit instructions. The optical density (OD) was measured at 450 nm using a MK3 microplate reader (Thermo Scientific). A standard curve was made and the serum levels of IL-18 were calculated.

### Index of immune organs

The thymus and spleen of each mouse were weighed, and the indices of immune organs were calculated using equation (2).

2 $${\rm{Index}}\,{\rm{of}}\,{\rm{immune}}\,{\rm{organ}}=\frac{{\rm{weight}}\,{\rm{of}}\,{\rm{immune}}\,{\rm{organ}}\,(\mathrm{mg})}{{\rm{body}}\,{\rm{weight}}\,({\rm{g}})}$$

### Phagocytic activity of monocyte/macrophage

The phagocytic activity of monocyte/macrophage in liver and spleen was assessed using a previously described method^39^. Briefly, after drug administration for ten days, India ink (0.01 mL/g body weight) was injected into the mouse via the vena caudalis. At 2 min (t1) and 10 min (t2) after injection, 30 μL of blood were collected via the eye orbit and immediately transferred to 3 mL of 0.1% Na_2_CO_3_ solution. The ODs at 600 nm were measured using a microplate reader. After the mice were sacrificed, the liver and spleen were removed and weighed. The index of carbon clearance (*K*) and the phagocytosis coefficient (*a*) were calculated using equations (3) and (4).

3 $$K=\frac{\mathrm{lg}\,O{D}_{1}-\,\mathrm{lg}\,O{D}_{2}}{{t}_{2}-{t}_{1}}$$

4 $$a=\frac{{\rm{body}}\,{\rm{weight}}}{{\rm{liver}}\,{\rm{weight}}+{\rm{spleen}}\,{\rm{weight}}}\times \sqrt[3]{K}$$

### Determination of serum hemolysin

Freshly prepared chicken blood was centrifuged for 10 min at 1500 rpm. After the supernatant and white blood cells were discarded, the remaining red blood cells were washed at least five times with physiological saline until the solution appeared transparent. The chicken red blood cells (CRBC) was diluted to 5% (v/v) using physiological saline and stored at 4 °C refrigerator before use.

On the fifth day of drug administration, 0.2 mL of 5% CRBC was intraperitoneally injected into each mouse for five continuous days. After last drug administration, blood was collected into an Eppendorf tube by orbital bleeding. The blood was centrifuged at 3,000 rpm for 15 min to isolate serum. The serum (10 μL) was diluted 100 times with physiological saline and then incubated with 0.5 mL of 5% CRBC and 0.5 mL of 10% guinea pig serum (as a hemolytic complement) at 37 °C for 1 h. The mixture was placed in refrigerator for 30 min to end the reaction. The supernatant was collected after centrifuging, and ODs of the supernatant were measured at 540 nm using a microplate reader.

### Cytotoxic activity of NK Cells

YAC-1 lymphoma cells were used as the target cells to determine the NK cell cytotoxicity. YAC-1 cells were cultured in RPMI-1640 medium and adjusted to a concentration of 4 × 10^5^ cells/mL. The spleens were isolated from mice under aseptic conditions and mashed in cold phosphate-buffered saline (PBS) through a sterilized strainer (200 mesh). After centrifuging at 1,000 rpm for 10 min, the spleen lymphocytes cells were resuspended in RPMI-1640 medium to a concentration of 2 × 10^7^ cells/mL. Mixtures of 100 μL spleen lymphocytes cells (effector cells) and 100 μL YAC-1 cells (target cells) were seeded in a U-bottom 96-well plate (effector: target = 50:1). The wells seeded with 100 μL YAC-1 cells and 100 μL cell culture medium served as a control to measure the spontaneous release of LDH. The wells seeded with 100 μL YAC-1 cells and 100 μL 1% NP-40 served as a control to measure the maximum release of LDH. After incubation at 37 °C in a 5% CO2 incubator for 4 h, the 96-well plate was centrifuged at 1500 r/min for 5 min and the supernatant was transferred to a flat-bottom 96-well plate. Subsequently, the cytotoxic activity levels of the NK cells in spleen lymphocytes were measured using a Lactate Dehydrogenase (LDH) Cytotoxicity Detection Kit. The plate was read at 490 nm in a microplate reader and the NK cell cytotoxicity (%) was calculated using equation (5).

5 $$ \% \,{\rm{of}}\,{\rm{damaged}}\,{\rm{cells}}=\frac{{\rm{experimental}}\,{\rm{release}}-{\rm{spontaneous}}\,{\rm{release}}}{{\rm{maximum}}\,{\rm{release}}-{\rm{spontaneous}}\,{\rm{release}}}\times 100$$

### Statistical Analysis

Data are presented as the mean ± S.D. Statistical analysis was performed using SPSS 13.0 software (IBM, Chicago, IL, USA). One-way ANOVA and t-tests were performed to determine the differences between the treated and control groups. Means were considered significantly different when *p*-value < 0.05.

### Data availability

All data generated or analysed during this study are included in this published article.
